# Rare adrenal gland incidentaloma: an unusual Ewing’s sarcoma family of tumor presentation and literature review

**DOI:** 10.1186/s12894-017-0217-3

**Published:** 2017-04-04

**Authors:** Hui Guo, Shuaiqi Chen, Shukun Liu, Kaixuan Wang, Erpeng Liu, Faping Li, Yuchuan Hou

**Affiliations:** 1 Department of Urology, First Hospital of Jilin University, Changchun, Jilin 130021 China; 2 Department of Urology, The First Affiliated Hospital of Xinxiang Medical University, Xinxiang, Henan 453100 China

**Keywords:** Ewing’s sarcoma family of tumor, Adrenal gland, Diagnosis, Treatment

## Abstract

**Background:**

Members of the Ewing’s sarcoma family of tumor (ESFT) are malignant neoplasms and rarely observed in the adrenal gland.

**Case presentation:**

We report an extremely exceptional case of ESFT rising from the adrenal gland in a 57-year-old Chinese man. The patient was hospitalized with abdominal swelling for 2 months. Computed tomography (CT) scan revealed a nearly-circular mass measuring about 8.1 × 10.6 cm in the right adrenal region. The patient underwent right adrenal resection. Histopathologic examination found the tumor was composed of small round blue cells forming typical Homer-Wright rosettes in focal area. The immunohistochemical analysis confirmed the case to be ESFT, which was positive for membranous CD99 and nuclear FLI-1. The patient was scheduled for four courses of large doses of chemotherapy and died for cancer metastasis one year later after surgery.

**Conclusions:**

Histopathological evidence of Homer-Wright rosettes and immunohistochemical markers positivity, such as CD99 and FLI-1, are valuable factors for ESFT diagnosis, although cytogenetic analysis is considered as the gold standard. Complete surgery is the treatment of choice for ESFT and adjuvant radiotherapy and combination chemotherapy can significantly improve the survival rate of postoperative patients.

## Background

The Ewing’s sarcoma family of tumor (ESFT) are rare aggressive malignancies and consist of Ewing’s sarcoma (ES) of bone, extraosseous Ewing’s, primitive neuroectodermal tumor (PNET), and Askin’s tumor [1, 2]. These distinct entities are characterized by common histopathological and immunohistochemical features, including a primitive undifferentiated small round blue cell associated to a variable level of palisading and rosette formation, as well as strongly positive for the cell surface glycoprotein CD99 [3–5]. The defining feature of the ESFT is a nonrandom chromosomal translocation and the most frequent is EWS-FLI1 fusion [6, 7]. These highly aggressive malignancies most commonly arise in the soft tissue or bone in adolescents and young adults [8]. Reports of cases arising from the adrenal gland are extremely rare. To the best of our knowledge, there are 32 cases in the English literatures [5, 9–30]. We report an additional ESFT case arising from the adrenal gland and discuss its clinical and histopathological characteristics, as well as unusual therapeutic strategies.

## Case presentation

**Fig. 1:**
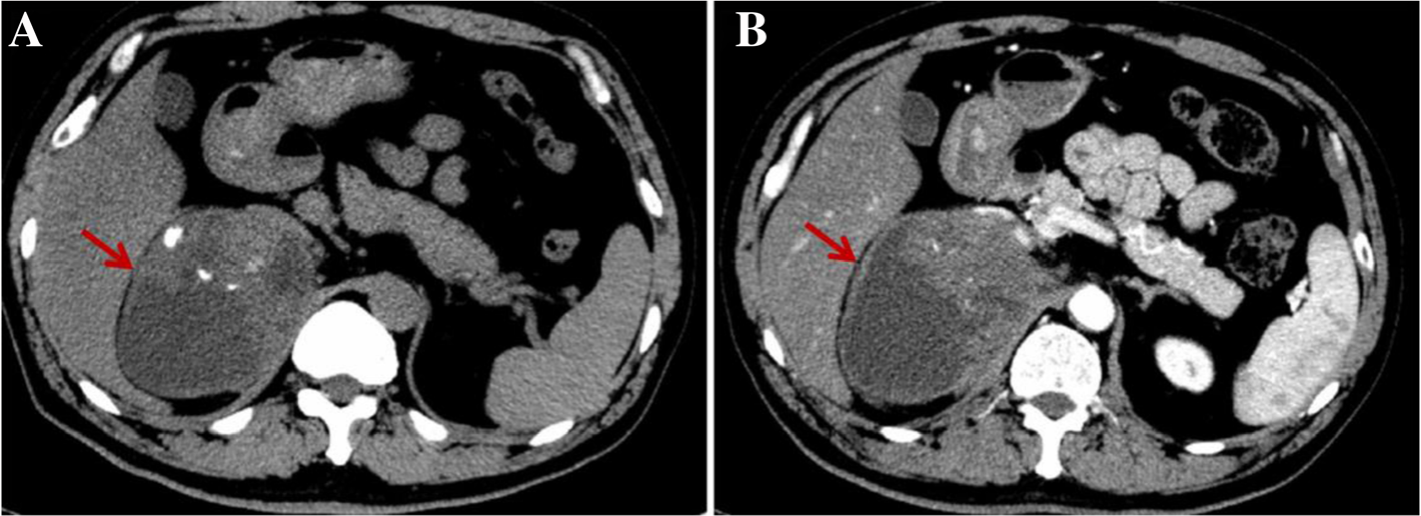
Abdominal computed tomography (CT) scan revealed a large mass (*arrow*) arising from the right adrenal gland (**a**). The lesion showed heterogeneous enhancement and relatively sharp margination (*arrow*) on Contrast-enhanced CT (**b**)

A 57-year-old man presented to the First Hospital of Jilin University (Changchun, China) with the main complaint of abdominal swelling for 2 months. In addition to the mild percussion pain in the right kidney region, no other symptoms were noted during a physical examination. His past medical history was unremarkable. Computed tomography (CT) scan of the abdomen revealed a nearly-circular mass measuring about 8.1 × 10.6 cm arising from the right adrenal gland (Fig. 1a). The CT also showed heterogeneous density, both solid and cystic components and calcification of the mass. The lesion showed heterogeneous enhancement and relatively sharp margination on Contrast-enhanced CT (Fig. 1b). Contrast-enhanced CT scan further defined the large mass was located between the liver and kidney with characteristics consistent with the soft tissue. Vena cava, right renal vein were compressed and displaced. No obvious metastasis was apparent.

**Fig. 2:**
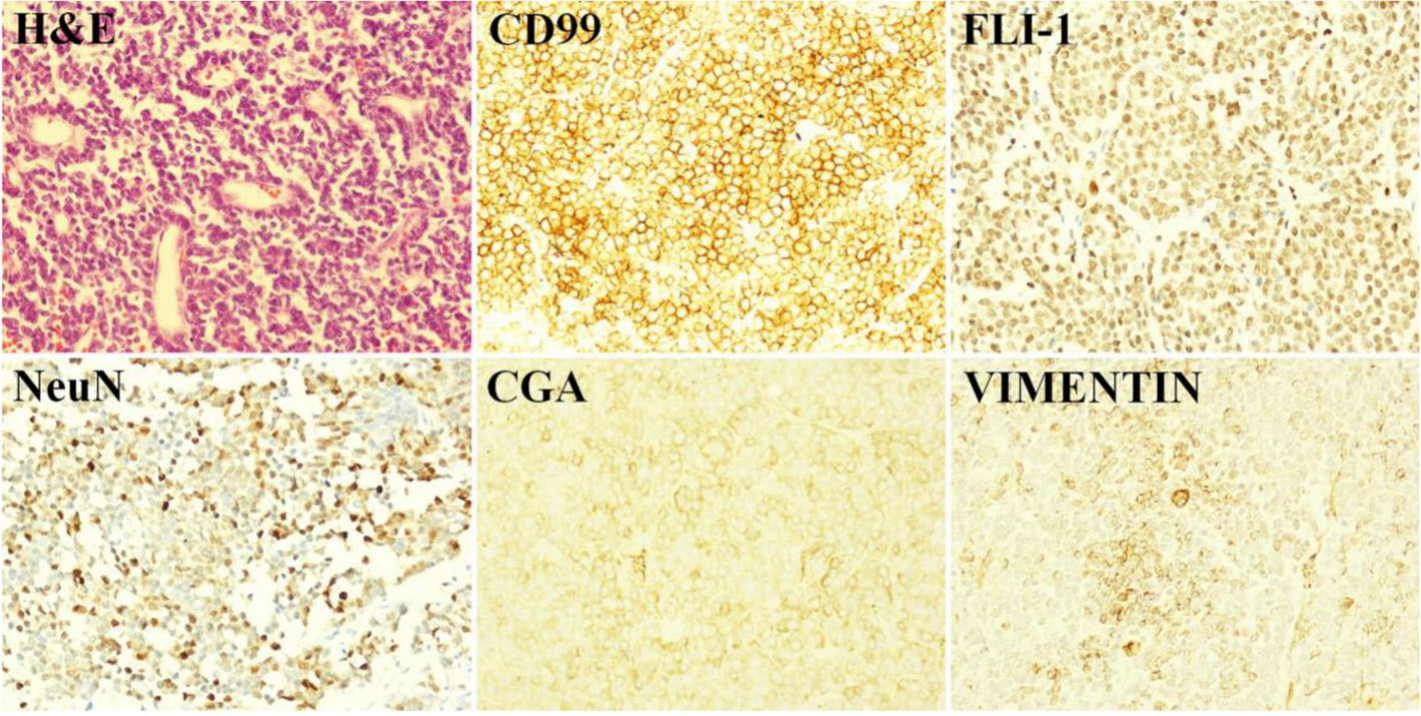
Histopathologic examination showed small round blue cells forming Homer-Wright-type rosettes (H&E, ×400). Immunohistochemical staining revealed the tumor cells were positive for CD 99, FLI-1, NeuN, CGA and VIMENTIN (original magnification × 400)

The patient underwent open surgery under general anesthesia. A 10.0 cm × 8.0 cm × 6.0 cm mass was found during laparotomy. The tumor was located above the left renal vein and the right renal vein without venous involvement. Due to firmly adhesion with the surrounding tissue, tumor dissection was difficult. Intraoperative blood loss was 800 mL and the tumor was completely removed eventually. Postoperative histopathology showed a monotonous population of small round blue cells with occasional Homer-Wright-type rosettes (Fig. 2). The results confirmed the diagnosis of PNET. The immunohistochemical staining was performed supporting the previous diagnosis, which was positive for CD99, FLI-1, NeuN, CGA and VIMENTIN (Fig. 2), while negative for EMA, SYN and LCA.

The patient was scheduled for adjuvant chemotherapy with adriamycin, cyclophosphamide, ifosfamide and etoposide. At his follow-up, 5 months after surgery, CT scan results demonstrated a metastatic lesion arising from the right abdominal wall. Unfortunately, the patient died for cancer metastasis one year later after surgery.

## Discussion

ESFT rising from the adrenal gland is extremely exceptional but malignant. Patients often present with tumor compression, flank pain or mass. However, its preoperative imaging diagnosis is difficult and histopathological and genetic tools are required for an accurate diagnosis.

Histopathologically, ESFT appear as immature or primitive small round blue cell tumors infiltrating the soft tissue or bone in a diffuse or lobular pattern. The tumor cells have round to oval nuclei with coarsely stippled chromatin and indistinct nucleoli. The scanty cytoplasm is pale or clear. In addition, these cells are often accompanied by hemorrhage and necrosis. ESFT are mainly represented by the existence of typical Homer-Wright-type rosette or other types of rosettes [17, 31].

Immunohistochemical markers such as CD99, FLI-1, HNK-1 and CAV-1 are commonly expressed in ESFT and provide valuable support to the definitive diagnosis. CD99, a 32-kDa cell surface glycoprotein, is encoded by the MIC2 gene and extremely sensitive for ESFT [4, 14]. The sensitivity is as high as 95% although the specificity is low [14, 31]. Its expression is also observed in T-lymphoblastic lymphoma, rhabdomyosarcoma, synovial sarcoma, and small cell anaplastic osteosarcoma [32–35]. ESFT can be potentially misdiagnosed based merely on expression of CD99. Even so, CD99 is still the most reliable immunohistochemical marker for ESFT. FLI-1, as well as HNK-1, appears reliable but less sensitive for ESFT than CD99 [4, 31]. All authors agree that both markers are expressed in various other round cell tumors [36]. CD99 and FLI-1 are mainly used for the diagnosis of ESFT and an immunohistochemical panel consisting at least these two makers is recommended [37–39]. CAV1, a membrane protein, its high expression is associated with the anchorage-independent growth [40, 41]. Express CAV1 have been shown to be more aggressive and metastatic [41]. CAV1 appears as a diagnostic immunohistochemical marker of ESFT being positive in CD99-negative cases [31]. In addition, markers of NSE, VIMENTIN, cytokeratin and S-100 have been detected in a subset of ESFT by immunohistochemistry.

At present, cytogenetic analysis is the “gold standard” for diagnosis of ESFT. Conventional tests are valuable to make the definitive diagnosis such as Southern blot, Northern blot analyses, FISH and RT-PCR [14, 39, 42]. The diagnosis of our case, ESFT rising from the adrenal gland, was not based on the cytogenetic findings. However, it was supported by the histopathological findings of poorly differentiated, small round blue cells forming typical Homer-Wright rosettes and the immunohistochemical findings of strongly positive for CD99, FLI-1 and negative for differentiation markers such as epithelial sufficiently.

ESFT is an aggressive malignancy with very poor prognosis [6]. Multimodality regimens including surgical resection, adjuvant chemotherapy and radiation therapy are often required [43]. Current surgical approaches include open, laparoscopic and robotic resection. The latter two are more difficult to perform because the large tumor is often accompanied by liquefaction and/or necrosis. Jacob Stephenson et al. [5] reported a ESFT arising from adrenal gland, during operation with the robotic assistance, the tumor capsule was ruptured, which may lead to metastasis and increase the dose of chemotherapy and radiotherapy. Hence, the surgical approach should be selected in accordance with patient’s condition.

Cooperative group studies have led to chemotherapy regimens using the same drugs (vincristine, doxorubicin, cyclophosphamide, ifosfamide, and etoposide), although the exact regimens differ in Europe and North America [2]. Only 16 cases of ESFT arising from the adrenal gland have been reported since 2011. Eleven of these sixteen patients received surgery. Nine received adjuvant chemotherapy and five received radiation treatment. Only two patients with small mass and no evidence of metastasis are alive and disease free. The two long-term survival of patients received multimodality regimens using a combination of complete surgery, as well as chemotherapy and radiotherapy (Table 1). We conclude that complete surgery is the treatment of choice for ESFT. Adjuvant chemotherapy and postoperative radiotherapy have shown significant improvements in survival. The tumor size and metastases are predictors for survival and effect prognosis obviously.

**Table 1:** Summary of Reported Cases of ESFT Rising from the Adrenal Gland (F: female; M: male; IVC: inferior vena cava; Surg: surgery; Chemo: chemotherapy; RTx: radiotherapy; NR: not recorded)

Case Report (Reference Number)	Age	Gender	Chief Complaint	Position	Tumor Size (cm)	Initial Infiltration or Metastasis	Treatment	Outcome at Time of Report
9	17	F	NR	NR	NR	Liver, lung, lymph node	Chemo + RTx	Dead
	8	M	NR	NR	NR	Bone, lung	Surg + chemo + RTx	Dead
	4	M	NR	NR	NR	Lung	Surg + chemo + RTx	Dead
10	46	F	NR	NR	NR	NR	NR	NR
	20	F	NR	NR	NR	NR	NR	NR
	48	F	NR	NR	NR	NR	NR	NR
11	32	F	Abdominal pain	Left	10	Liver	Surg + adjuvant chemo	Dead
12	57	M	Lower extremity pain, edema	Right	15	None	Surg	NR
13	11	M	Abdominal tumor	Right	13	Peritoneum	Surg + chemo + RTx	Dead
14	28	F	Recurrent mass	Right	10	Lung	Surg + chemo	NR
15	25	F	Abdominal pain	Left	15.2	IVC, lung	NR	NR
	24	F	Flank pain	NR	8.4	Supraclavicular lymph node	NR	NR
16	53	F	Adrenal tumor	Right	3	None	Surg	Alive
17	30	M	NR	Right	12	IVC tumor embolus	Surg + RTx	Dead
	21	F	NR	Left	10	Liver	None	Dead
	24	F	NR	Left	9	Pelvic lymph node	Surg + chemo	Metastasis
	22	M	NR	Left	17	IVC tumor embolus	Surg + chemo	Local recurrence
18	20	F	Flank pain, anorexia, weight loss	Right	Large	Lung	Neo-adjuvant chemo	Unknow
5	17	F	Flank pain	Right	5	None	Surg + adjuvant chemo + RTx	Alive
19	26	F	Flank pain	Left	Large	IVC tumor thrombus	Surg + chemo + RTx	Alive
20	17	M	Swelling, abdominal pain	Right	21.3	Liver, lung	Systemic chemotherapy	Alive
21	17	F	Abdominal Pain, fever	Left	15	None	Surg + adjuvant chemo + RTx	Recurrence
22	26	F	Flank pain	Left	11.3	IVC tumor thrombus	Surg + adjuvant chemo + RTx	Alive
23	63	M	None	Left	3.2	None	Surg + adjuvant chemo	Alive
24	40	F	Abdominal pain, swelling, respiratory distress	Left	14.6	Retroperitoneal muscles	Surg + adjuvant chemo	Alive
25	37	F	Loin pain	Left	8	Kidney	Surg	Alive
26	26	M	None	Right	8	None	None	Dead
27	37	F	Flank pain, abdominal pain	Left	12	Crus of diaphragm, kidney	Surg + adjuvant chemo	Alive
28	17	F	Abdominal pain	Left	3.3	None	Surg + adjuvant chemo + RTx	Alive
29	23	M	Flank pain, weight loss	Right	15	Kidney, head of pancreas, liver	None	Unknow
	27	M	Pain	Right	NR	Kidney, liver, pancreas	Chemo	Dead
30	48	F	Abdominal pain, swelling	Left	12	None	Surg	Recurrence
Present case	57	M	Swelling	Right	10.6	None	Surg + adjuvant chemo	Dead

## Conclusion

ESFT rising from the adrenal gland is a rare clinical entity. Histopathological evidence of Homer-Wright is crucial for ESFT diagnosis. The neural markers, such as CD99, FLI-1, HNK-1 and CAV-1, may play a valuable role in the immunohistochemical diagnosis of ESFT. The definitive diagnosis of ESFT requires a combination of immunohistochemical examination, as well as histopathologic evaluation, although the “gold standard” will obviously remain cytogenetic analysis. Complete surgery is the treatment of choice for ESFT. Adjuvant chemotherapy and postoperative radiotherapy have shown significant improvements in survival. The tumor size and metastases are predictors for survival and effect prognosis obviously.

## Abbreviations

CAV-1: Caveolin-1
CD99: Cluster of differentiation 99
CGA: Chromogranin A
CT: Computed tomography
ES: Ewing’s sarcoma
ESFT: Ewing’s sarcoma family of tumor
LCA: Leukocyte common antigen
NSE: Neuron-specific enolase
PNET: Primitive neuroectodermal tumor
SYN: Synaptophysin
